# Seasonal Determination of Antibiotic-Resistant Microorganisms and Ciprofloxacin Residues in Pork and Chicken Meats Collected from Fresh Markets in Chiang Mai, Northern Thailand

**DOI:** 10.3390/foods14020174

**Published:** 2025-01-08

**Authors:** Sirikwan Dokuta, Sumed Yadoung, Sayamon Hongjaisee, Phadungkiat Khamnoi, Sirinya Manochomphu, Bajaree Chuttong, Surat Hongsibsong

**Affiliations:** 1 School of Health Sciences Research, Research Institute for Health Sciences, Chiang Mai University, Chiang Mai 50200, Thailand; sirikwan_d@cmu.ac.th (S.D.); sayamon.ho@cmu.ac.th (S.H.); 2 Environmental Sciences Program, Faculty of Sciences, Chiang Mai University, Chiang Mai 50200, Thailand; sumed_y@cmu.ac.th; 3 Diagnostic Laboratory, Microbiology Unit, Maharaj Nakorn Chiang Mai Hospital, Faculty of Medicine, Chiang Mai University, Chiang Mai 50200, Thailand; phadungkiat.k@cmu.ac.th (P.K.); sirinya.m@cmu.ac.th (S.M.); 4 Meliponini and Apini Research Laboratory, Department of Entomology and Plant Pathology, Faculty of Agriculture, Chiang Mai University, Chiang Mai 50200, Thailand; bajaree.c@cmu.ac.th; 5 Environmental, Occupational Health Sciences and NCD Center of Excellence, Research Institute for Health Sciences, Chiang Mai University, Chiang Mai 50200, Thailand

**Keywords:** fluoroquinolone, antibiotic residue, ciprofloxacin, antibiotic resistance, ESBL, chicken, pork, microorganism, Chiang Mai, Thailand, seasonal

## Abstract

Antibiotic-resistant bacteria are usually found in food-producing animals worldwide. Ciprofloxacin, an antibiotic, can lead to antibiotic residues in food products, posing health risks to consumers and contributing to the development of antimicrobial resistance. Foodborne illnesses occur when adequate attention is not paid to food hygiene and safety, raising the potential for resistant bacteria to spread to humans through the food chain. This study aims to determine the presence of antibiotic-resistant organism contamination and ciprofloxacin residue in raw pork and chicken. Forty-three pork and 33 chicken meat samples were collected from fresh markets in Chiang Mai, Thailand. Antibiotic-resistant organisms were detected by microbial culture and identified by MALDITOF-MS. The antimicrobial sensitivity tests were used to confirm antibiotic resistance. The ciprofloxacin was detected by using an immunochromatographic-based test kit for screening. The results found Extended Spectrum β-lactamase (ESBL)-producing *E. coli* and *K. pneumoniae* were detected at 46.51% and 9.30% in pork and 69.70% and 6.06% in chicken meat samples, respectively. Moreover, ciprofloxacin residues were detected in nine samples (11.84%). Based on this study’s findings, the people who are involved in the food chain must be concerned about food safety and food hygiene.

## 1. Introduction

Antibiotic resistance has become one of the most critical global health challenges over the past decade, causing significant impacts on national and international public health systems [1]. Historically, antibiotics have been utilized not only for medical treatments but also as growth promoters in agriculture and livestock, potentially resulting in environmental contamination of water and soil [2,3]. These contaminations can have adverse effects on various ecological systems, affecting different trophic levels and contributing to the spread of resistant bacteria across different ecosystems [3].

The misuse and overuse of antibiotics in both human and veterinary contexts have accelerated the emergence of antibiotic-resistant pathogens [4,5]. In human health, the consumption of food and water contaminated with antibiotic residues can contribute to the increasing prevalence of resistant bacterial strains. Such strains can persist in the environment, spreading through food chains and contaminating surfaces, including those in healthcare settings [6,7,8,9]. Contaminated water bodies, often impacted by fecal residues, are another source of infections associated with resistant bacteria [10]. This “farm-to-fork” transmission pathway highlights the significant role that animal-derived food products play in the dissemination of antibiotic resistance, as direct contact with infected water or consumption of contaminated foods can expose individuals to resistant pathogens [11,12].

In livestock production, the routine use of antibiotics is a major contributor to the emergence of antibiotic-resistant bacteria in food animals [13]. Since certain antibiotic classes are shared between human and veterinary medicine, monitoring the transfer of resistant bacteria from animals to humans at each stage of the transmission pathway is essential [14]. Foodborne illnesses often arise when hygiene and safety protocols are not strictly adhered to, allowing bacteria to spread through the food chain and reach consumers [15,16]. Contaminated meat can carry bacteria resistant to multiple drugs, posing a severe threat to human health [17].

Previous studies have indicated that antibiotic-resistant pathogens are particularly prevalent in poultry, pig, and other meat animals [18,19,20]. During the slaughtering, processing, and transportation phases, resistant bacteria from the gut can contaminate meat products, potentially infecting consumers [21,22,23]. In Thailand, common antibiotic-resistant bacteria include Methicillin-Resistant *Staphylococcus aureus* (MRSA), Extended Spectrum β-lactamase (ESBL)-producing Gram-negative bacilli, Carbapenem-resistant *Enterobacteriaceae* (CRE), and Vancomycin-resistant Enterococci (VRE). These resistant strains have been reported not only in hospitals but also in food animals, underscoring the need for vigilance [24].

Nowadays, the presence of antibiotic residues in food products, particularly meats, poses significant public health concerns due to the potential for antibiotic resistance and foodborne illnesses [25,26,27].

Fluoroquinolones, a class of broad-spectrum antibiotics such as ciprofloxacin, norfloxacin, and enrofloxacin, are commonly used in livestock production to prevent and treat bacterial infections [28,29]. In particular, ciprofloxacin exhibits strong bactericidal effect activity. It demonstrates effectiveness against *Enterobacter*, *Pseudomonas aeruginosa*, *Haemophilus influenzae*, *Neisseria gonorrhoeae*, *Streptococcus*, *Legionella*, and *S. aureus*. Its antibacterial efficacy is two to four times greater than that of norfloxacin and enoxacin against nearly all bacteria. However, its widespread use raises concerns about residues that may persist in animal-derived food products, which can contribute to the development of antibiotic-resistant bacteria and pose risks to consumers [30].

Under the development model of precision medicine, new requirements are put forward for therapeutic drug monitoring. Rapidity, simplicity, and accuracy of drug concentration monitoring are the trends of its clinical application [31].

The immunoassay for detecting antibiotic residues offers convenience, whereas alternative methods present limitations, including a reduced number of detectable drugs, high costs of instruments, and time-intensive processes [32]. Lateral flow immunoassay (LFIA) has been utilized for semi-quantitative, quantitative, and environmental monitoring purposes. A traditional LFIA consists of a strip made of a carrier substance that holds a dry reagent, which becomes activated upon contact with fluid samples.

Screening assay testing is frequently utilized as a semi-quantitative approach to detect the presence of analytes in the sample. Screening represents a straightforward, swift, and economical assay approach that ensures high reproducibility and dependable outcomes [33].

Nevertheless, a previous report found the relation between ciprofloxacin resistance and Extended Spectrum β-lactamase (ESBL), so the residues of fluoroquinolones, especially ciprofloxacin in animal-based food, are required to be monitored and assessed [30].

To safeguard public health, continuous monitoring of antibiotic-resistant bacteria in food products is crucial [26]. Early detection of foodborne pathogens in livestock products can prevent outbreaks and control the spread of resistance [34]. Therefore, this study investigates the prevalence of antibiotic-resistant organism contamination and determines ciprofloxacin residue by using Lateral flow immunoassay in pork and chicken sold at fresh markets in Chiang Mai, Northern Thailand, emphasizing the importance of food safety measures and the need for surveillance to reduce the risks posed by antibiotic-resistant bacteria in animal-derived foods.

## 2. Materials and Methods

### 2.1. Study Design

This study collected meat samples from three fresh markets in Chiang Mai Province, Thailand, from September 2023 to February 2024. These samples were used to detect antibiotic-resistant organisms and ciprofloxacin residue, respectively. The sample collection was separated into three rounds. Round 1 was collected during September–October 2023; Round 2 was collected during November–December 2023, and Round 3 was collected during January–February 2024. The weather conditions during our sample collection period were rainy/wet during September–October (Round 1), hot and dry during November–December (Round 2), and cold during January–February (Round 3) in this area.

### 2.2. Sample Collection

Raw chicken wings and pork shoulder meat samples were collected from three markets in Chiang Mai, Thailand. These meat samples were randomly purchased from the meat shops in wet markets (one sample per shop) and were kept in clean plastic and zip lock bags, labeled, and stored in a container with the ice pack to control the temperature between 20–25 °C, and then were transported to the laboratory as soon as possible or within 8 h. The total number of meat samples collected was 76 samples (43 pork shoulder meat and 33 chicken wing samples).

### 2.3. Detection of Antibiotic-Resistant Microorganisms

#### 2.3.1. Bacterial Culture Test (Culture-Based Method)

Sterile amies swab was used to swab on the surface of each 100 g of raw meat sample (one by one for each sample) and then was brought to swab in a small circular pattern on the four types of CHROMID^®^ selective agar plates (bioMerieux SA, Marcy l’Etoile, France). After that, the sterile loop was used to streak back and forth in a zigzag pattern on the surface of each agar plate for three plains to spread the sample from a circular area. Finally, these agar plates were incubated at 35 ± 2 °C for 18–24 h. The same procedure was performed for all CHROMID^®^ ESBL, CHROMID™ CARBA, CHROMID^®^ MRSA SMART, and CHROMID™ VRE agar plates for the detection of ESBL-producing bacteria, Carbapenem-resistant *Enterobacteriaceae* (CRE), Methicillin-resistant *Staphylococcus aureus* (MRSA) and Vancomycin-resistant Enterococci (VRE) organism, respectively.

#### 2.3.2. Bacterial Isolation and Identification Test

For bacterial isolation, a sterile loop was used to pick up the suspected colony. The colony from the CHROMID^®^ ESBL and CHROMID™ CARBA agar plates were isolated on MacConkey agar, whereas the colony from the CHROMID^®^ MRSA SMART and CHROMID™ VRE agar plates were isolated on Phenylethyl alcohol (PEA) agar plate. After that, these plates were incubated at 35 ± 2 °C for 18–24 h.

For bacterial identification, the isolated single colony of suspected bacteria was identified as the organism strain by using the automated Matrix-assisted laser desorption-ionization time of flight mass spectrometry (MALDI-TOF MS), VITEK^®^ MS (bioMerieux, Marcy l’Etoile, France) machine.

#### 2.3.3. Antimicrobial Resistant Drug Susceptibility Test (Disk Diffusion Method Follow CLSI)

The standardized antimicrobial sensitivity test was performed on Mueller-Hinton agar (MHA) plates by disc diffusion Kirby-Bauer technique with 0.5 McFarland turbidity standard methods, and results were interpreted according to the Standards for Antimicrobial Susceptibility of the Clinical Laboratory Standards Institute (CLSI) protocol [35].

Briefly, well-isolated bacterial colonies were selected from cultured agar plates and were suspended in broth culture to a 0.5 McFarland standard. A sterile swab was dipped into the standardized suspension of bacteria, and excess fluid was removed by pressing and rotating the swab firmly against the inside of the tube above the fluid level. This swab was used to streak on the whole surface of the MHA plate, and then antibiotic discs appropriate for organisms were placed onto the surface of the MHA plate. Plates were incubated at 37 ± 2 °C for 18–24 h. The zones of inhibition were measured to the nearest millimeter at the back of the inverted culture plate. The measurement results were compared with a standard chart adopted by CLSI to determine susceptibility or resistance.

The antimicrobial susceptibility was performed on the suspected isolated colonies using four antibiotic discs: Ceftazidime (CAZ30), Cefotaxime (CTX30), Ceftazidime/Clavulanic acid (CAC 30/10), and Cefotaxime/Clavulanic acid (CEC30/10) was used to confirm the extended-spectrum β-lactamase (ESBL)-producing bacteria.

*Escherichia coli* ATCC^®^ 25922, *Escherichia coli* ATCC^®^ 35218, *Staphylococcus aureus* ATCC^®^ 25923, *Staphylococcus aureus* ATCC^®^ 29213 and *Pseudomonas aeruginosa* ATCC^®^ 27853 were used for quality control.

### 2.4. Determination of Ciprofloxacin Residue

The ciprofloxacin test kit (Lateral flow immunoassay, Wanlian, Guangzhou WanLian Bio-Technology Co., Ltd., Guangzhou, China) was used to determine the residue. The test kit is suitable for rapid qualitative detection of ciprofloxacin in meat, livestock, poultry, and other tissues. The detection limit of ciprofloxacin was 100 μg/kg. The determination process followed the product’s protocol.

### 2.5. Statistical Analysis

To determine significant differences between seasonal antibiotic resistance detected, the One-way ANOVA test was used to assess them, and a *p*-value of less than 0.05 was considered significant.

## 3. Results

### 3.1. Antibiotic-Resistant Microorganisms Detection

The detection results of antibiotic-resistant organisms in raw meat samples with pork shoulder and chicken wings meat purchased from three fresh markets in Chiang Mai province, Thailand, by using microbial culture and identification are presented in Table 1, Table 2, Table 3 and Table 4.

Among pork samples, this study found 2, 1, 9, and 19 raw pork meat samples provided none of the microorganisms grew on CHROMID^®^ ESBL, CHROMID™ CARBA, CHROMID^®^ MRSA SMART, and CHROMID™ VRE agar plates, respectively. Whereas for raw chicken meat samples, 1, 8, and 15 samples found no growth of organisms on CHROMID^®^ ESBL, CHROMID^®^ MRSA SMART, and CHROMID™ VRE agar plates, respectively.

For the results of antibiotic-resistant microorganisms isolated from CHROMID^®^ ESBL agar plates, the results of pork and chicken meat samples collected in Rounds 1–3 are shown in Table 1. Of pork meat sample results, ESBL-producing Gram-negative bacilli bacteria with specific-colored colonies were detected from samples collected in Round 1 at 78.57% (11/14) for *Escherichia coli* and 14.29% (2/14) for *Klebsiella pneumoniae*. ESBL-producing *E. coli* and *K. pneumoniae* were detected at 40.00% (6/15) and 13.33% (2/15), respectively, from samples collected in Round 2. ESBL-producing *E. coli* and *K. pneumoniae* were detected at 21.43% (3/14) and 7.14% (1/14), respectively, in the Round 3 collection.

In addition, for the results of ESBL-producing microorganisms detected in chicken meats, ESBL-producing *E. coli* was detected at 72.73% (8/11), and ESBL-producing *K. pneumoniae* was detected at 9.09% (1/11) from samples collected in Round 1. For Round 2 collection, ESBL-producing *E. coli* and *K. pneumoniae* were detected at 81.82% (9/11) and 9.09% (1/11), respectively. In contrast, only ESBL-producing *E. coli* was detected from a sample collected in Round 3, which was 54.55% (6/11).

Aside from the ESBL-producing microorganisms detected, other organisms were detected from pork and chicken meat samples with colorless colonies detected on CHROMID^®^ ESBL agar plates. Among pork samples, *A. punctata* (*caviae*) was the most isolated at 28.57% (4/14) from samples collected in Round 1, followed by *A. ursingii* and *A. guillouiae* at 21.43% (3/14), *A. nosocomialis*, *P. fragi,* and *P. putida* at 14.29% (2/14).

While for pork samples collected in Round 2, *C. indologenes* was the most isolated at 40.00% (6/15), followed by *A. ursingii* at 33.33% (5/15), *A. punctata* (*caviae*), and *P. putida* at 20.00% (3/15); *P. fluorescens* and *S. fonticola* at 13.33% (2/15).

Whereas for pork samples collected in Round 3, *P. putida* was the most isolated at 28.57% (4/14), followed by *C. freundii* at 21.43% (3/14), *A. punctata* (*caviae*), and *P. fluorescens* at 14.29% (2/14).

Of other microorganisms detected from chicken meat samples, *A. punctata* (*caviae*), *A. salmonicida*/*bestiarum,* and *P. putida* were the most isolated at 36.36% (4/11) from samples collected in Round 1, followed by *A. media*, *Pseudomonas* spp., and *S. fonticola* at 18.18% (2/11).

For chicken samples collected in Round 2, *A. punctata* (*caviae*), *Myroides* spp., and *P. putida* were the most isolated at 27.27% (3/11), followed by *A. pittii*, *A. salmonicida/bestiarum*, *C. indologenes*, *P. fragi,* and *P. fluorescens* at 18.18% (2/11).

For chicken samples collected in Round 3, *A. salmonicida*/*bestiarum* was most isolated at 36.36% (4/11), followed by *E. americana*, *P. fragi*, *P. putida,* and *S. maltophilia* at 18.18% (2/11).

In addition, other organisms detected in pork and chicken meats are named in Table 1.

**Figure 1:**
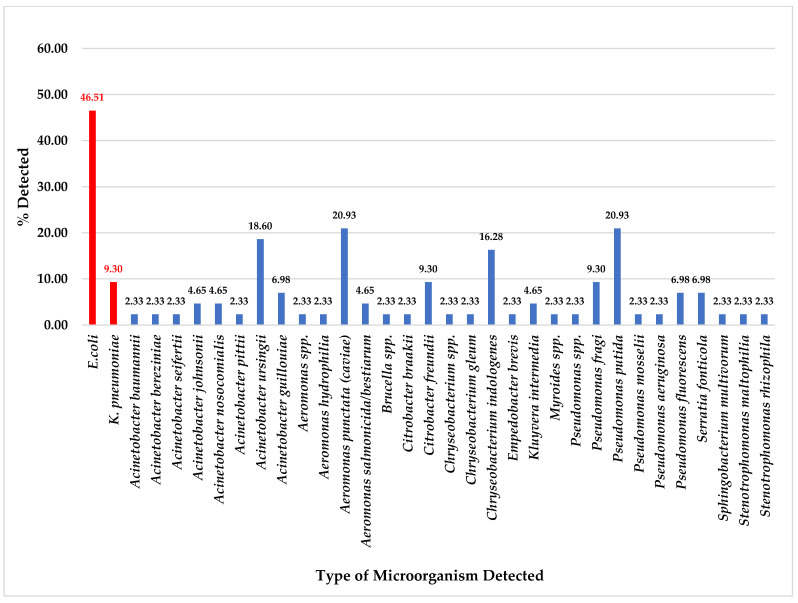
Total percentage of microorganisms detected in pork meat samples collected from fresh markets in Chiang Mai Province (September 2023–February 2024, *n* = 43). Microorganisms were isolated by CHROMID^®^ ESBL agar plates. Red color bars indicate ESBL-producing organisms while blue color bars indicate non ESBL- producing organisms.

Of the total 43 collected pork meat samples, ESBL-producing bacteria were detected in 20 (46.51%) samples for *E. coli* and 4 (9.30%) samples for *K. pneumoniae*. Other microorganisms detected on CHROMID^®^ ESBL agar plates, *A. punctata* (*caviae*) and *P. putida,* were most detected at 20.93% (9/43), followed by *A. ursingii* at 18.60% (8/43), *C. indologenes* at 16.28% (7/43), *C. freundii* and *P. fragi* at 13.33% (4/43) (Figure 1). Nevertheless, some organisms were also detected in pork meat samples as equal to or less than 6.98% (3/43), as shown in Figure 1.

**Figure 2:**
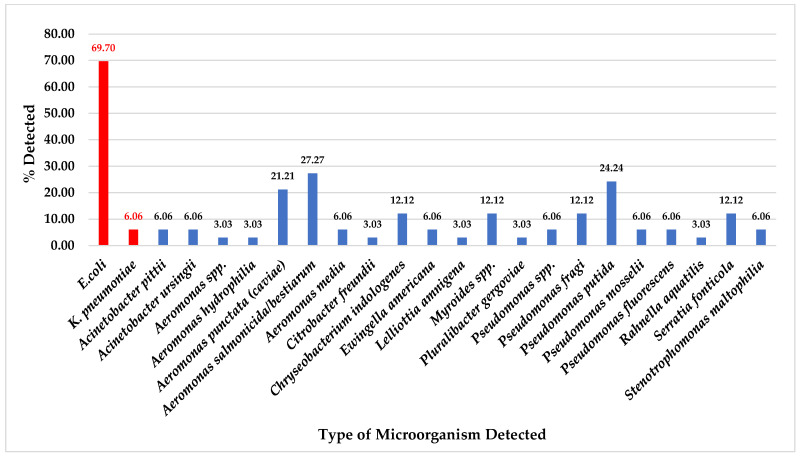
Total percentage of microorganisms detected in chicken meat samples collected from fresh markets in Chiang Mai Province (September 2023–February 2024, *n* = 33). Microorganisms were isolated by CHROMID^®^ ESBL agar plates. Red color bars indicate ESBL-producing organisms while blue color bars indicate non ESBL- producing organisms.

While a total of 33 chicken meat samples were collected, ESBL-producing bacteria were detected at 69.70% (23/33) for *E. coli* and 6.06% (2/33) for *K. pneumoniae*. At the same time, 27.27% (9/33) of *A. salmonicida/bestiarum*, 24.24% (8/33) of *P. putida,* and 21.21% (7/33) of *A. punctata* (*caviae*) were detected on CHROMID^®^ ESBL agar plates. Moreover, other organisms were also detected in chicken meat samples as equal to or less than 12.12% (4/43), as shown in Figure 2.

For the results of microorganisms isolated from CHROMID^TM^ CARBA agar plates, this study found no antibiotic-resistant organism with specific-colored colonies detected in pork and chicken meat samples. Nevertheless, other organisms with colorless colonies were isolated from these samples collected in Rounds 1–3 (Table 2). Among pork samples collected in Round 1, *A. sobria* was the most isolated on CHROMID^TM^ CARBA agar plates at 78.57% (11/14), followed by *P. putida at* 35.71% (5/14), *Aeromonas* spp., and *P. fluorescens at* 28.57% (4/14), and *A. veronii*, *Pseudomonas* spp. and *P. fragi at* 14.29% (2/14).

Of pork samples collected in Round 2, *A. veronii* was the most isolated on CHROMID^TM^ CARBA agar plates at 73.33% (11/15), followed by *P. putida* at 46.67% (7/15), *S. multivorum* at 40.00% (6/15), *S. maltophilia* at 26.67% (4/15), and *A. salmonicida/bestiarum*, *A. sobria* and *P. fragi* at 20.00% (3/15).

From pork samples collected in Round 3, *A. veronii*, *A. sobria,* and *P. putida* were the most isolated on CHROMID^TM^ CARBA agar plates at 35.71% (5/14). This was followed by *P. fragi* and *S. multivorum* at 21.43% (3/14) and *P. mosselii* and *S. liquefaciens* at 14.29% (2/14).

Whereas for the results of microorganisms detected in chicken meat samples which were isolated from CHROMID™ CARBA agar plates, *A. sobria* was the most isolated from samples collected in Round 1 at 81.82% (9/11), followed by *P. putida* at 63.64% (7/11), *Pseudomonas* spp. and *P. fluorescens at* 27.27% (3/11), and also detected *Aeromonas* spp., *A. veronii*, *P. fragi,* and *P. mosselii at* 18.18% (2/11).

For chicken samples collected in Round 2, *A. veronii* was the most isolated on CHROMID™ CARBA agar plates at 63.642% (7/11), followed by *P. putida at* 54.55% (6/11), *A. sobria* at 27.27% (3/11), *A. salmonicida*/*bestiarum*, *Pseudomonas* spp. and *P. fluorescens at* 18.18% (2/11), and also detected *C. indologenes*, *P. fragi* and *P. mosselii* and *S. maltophilia at* 9.09% (1/11).

For chicken samples collected in Round 3, *A. veronii* was the most isolated on CHROMID™ CARBA agar plates at 63.642% (7/11), followed by *A. salmonicida*/*bestiarum*, *P. putida*, and *P. fluorescens* at 36.36% (4/11), *A. sobria* at 27.27% (3/11), *P. fragi* and *S. maltophilia at* 18.18% (2/11), and also detected *A. pittii*, *P. chlororaphis*, *P. aeruginosa*, *S. liquefaciens*, *S. multivorum*, and *S. rhizophila* at 9.09% (1/11).

**Figure 3:**
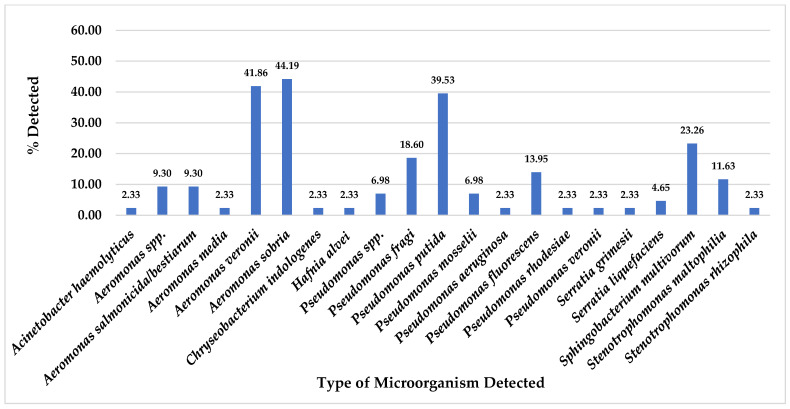
Total Percentage of Microorganisms Detected in Pork Meat Samples from Fresh Markets in Chiang Mai Province (September 2023–February 2024, *n* = 43). Microorganisms were isolated using CHROMID™ CARBA agar plates.

Of total 43 pork meat collected samples, this study found *A. sobria* was the most detected on CHROMID™ CARBA agar plates at 44.19% (19/43), followed by *A. veronii* at 41.86% (18/43), *P. putida* 39.53% (17/43), *S. multivorum* at 23.26% (10/43), *P. fragi* at 18.60% (8/43), *P. fluorescens* at 13.95% (6/43) and *S. maltophilia* at 11.63% (5/43). Nevertheless, some organisms were also detected in pork meat samples at equal to or less than 9.30% (4/43), as shown in Figure 3.

**Figure 4:**
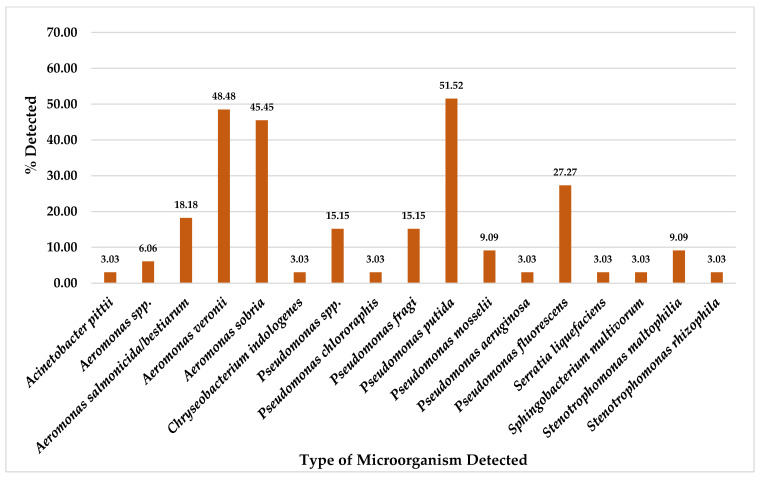
Total Percentage of Microorganisms Detected in Chicken Meat Samples from Fresh Markets in Chiang Mai Province (September 2023–February 2024, *n* = 33). Microorganisms were isolated using CHROMID™ CARBA agar plates.

For a total of 33 chicken meat collected samples, this study found *P. putida* was the most detected on CHROMID™ CARBA agar plates at 51.52% (17/33), followed by *A. veronii* at 48.48% (16/33), *A. sobria* at 45.45% (15/33), *P. fluorescens* at 27.27% (9/33), *A. salmonicida*/*bestiarum* at 18.18% (6/33), and *Pseudomonas* spp. and *P. fragi* at 15.15% (5/33). Furthermore, other organisms were also detected in chicken meat samples at equal to or less than 9.09% (3/33), as shown in Figure 4.

For the microorganisms isolated from CHROMID^®^ MRSA SMART agar plates, this study found no antibiotic-resistant organism detected from pork and chicken meat samples collected in Rounds 1–3. However, other organisms with colorless colonies were isolated from these samples; all results are shown in Table 3.

Of pork meat samples collected in Round 1, *M. caseolyticus* and *V. fluvialis* were the most isolated organisms at 21.43% (3/14), followed by *R. kristinae* at 14.29% (2/14).

While from pork samples collected in Round 2, *R. kristinae* was the most isolated organism at 60.00% (9/15), followed by *M. caseolyticus* at 20% (3/15), *M. liquefaciens* and *V. fluvialis at* 13.33% (2/15), *Bacillus cereus* group, and *C. neteri at* 6.67% (1/15).

For pork samples collected in Round 3, *E. faecalis* and *R. kristinae* were the most isolated organisms at 28.57% (4/14), followed by *M. caseolyticus* and *V. fluvialis* at 21.43% (3/14), *C. divergens*, *L. garvieae*, *S. sciuri,* and *W. paramesenteroides* at 14.29% (2/14), and also detected *C. gilardii*, *M. liquefaciens*, *M. morganii*, *S. saprophyticus* and *V. harveyi* at 7.14% (1/14).

Whereas of chicken meat samples, for Round 1 collection, *M. caseolyticus* was the most isolated organism from CHROMID^TM^ MRSA SMART agar plates at 18.18% (2/11), followed by *L. raffinolactis*, *M. liquefaciens,* and *V. fluvialis* at 9.09% (1/11).

Of chicken samples collected in Round 2, *M. caseolyticus* was the most isolated organism at 36.36% (4/11), followed by *C. davisae*, *M. liquefaciens* and *R. kristinae* at 18.18% (2/11), and also detected *C. pseudodiphtheriticum*, *E. faecalis* and *L. raffinolactis* at 9.09% (1/11).

From chicken samples collected in Round 3, *E. faecalis* was the most isolated organism at 36.36% (4/11), followed by *M. caseolyticus* at 27.27% (3/11), *S. sciuri* at 18.18% (2/11), and also detected *C. neteri*, *E. faecium*, *M. liquefaciens*, *M. oxydans*, *R. kristinae*, *S. kloosii*, *S. saprophyticus,* and *V. fluvialis* at 9.09% (1/11).

**Figure 5:**
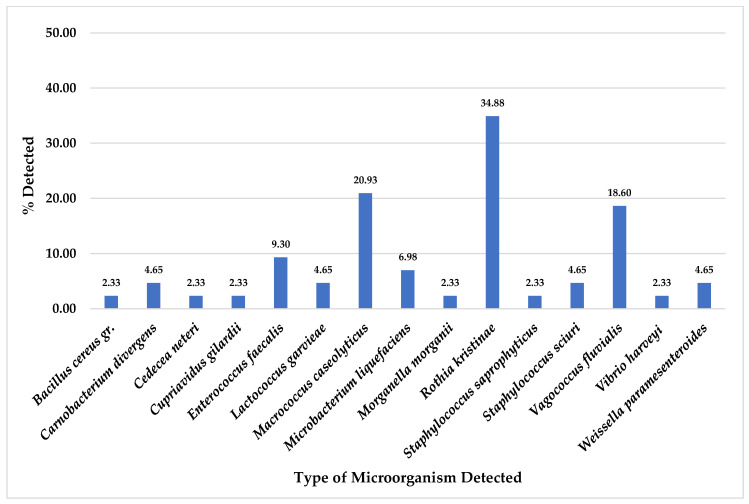
Total Percentage of Microorganisms Detected in Pork Meat Samples from Fresh Markets in Chiang Mai Province (September 2023–February 2024, *n* = 43). Microorganisms were isolated using CHROMID^®^ MRSA SMART agar plates.

For totally 43 pork meat collected samples, this study found *R. kristinae* was the most detected on CHROMID^®^ MRSA SMART agar plates at 34.88% (15/43), followed by *M. caseolyticus* at 20.93% (9/43), *V. fluvialis* at 18.60% (8/43), *E. faecalis* at 9.30% (4/43) and *M. liquefaciens* at 6.98% (3/43) (Figure 5). Moreover, other organisms were also detected in pork meat samples at equal or less than 4.65% (2/43), as shown in Figure 5.

**Figure 6:**
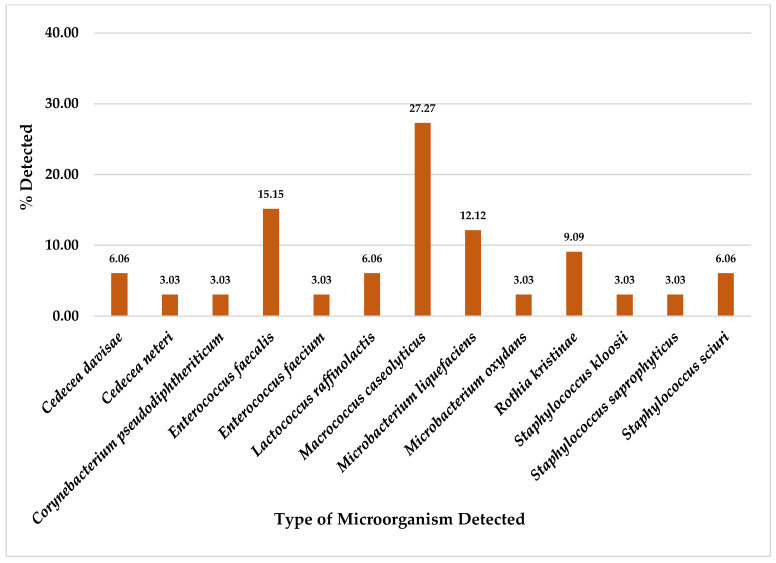
Total Percentage of Microorganisms Detected in Chicken Meat Samples from Fresh Markets in Chiang Mai Province (September 2023–February 2024, *n* = 33). Microorganisms were isolated using CHROMID^®^ MRSA SMART agar plates.

Of a total of 33 chicken meat collected samples, this study found *M. caseolyticus* was the most detected on CHROMID^®^ MRSA SMART agar plates at 27.27% (9/33), followed by *E. faecalis* at 15.15% (5/33), *M. liquefaciens* at 12.12% (4/33) and *R. kristinae* at 9.09% (3/33). Furthermore, other organisms were also detected in chicken meat samples at equal or less than 6.06% (2/33), as shown in Figure 6.

Among the microorganisms isolated from CHROMID™ VRE agar plates, this study found no antibiotic-resistant organism was detected in pork and chicken meat samples collected in Rounds 1–3. Nevertheless, other organisms were detected in these samples, and the results are shown in Table 4.

Of the results of pork meat samples, *C. indologenes*, *Myroides* spp., and *M. marinus* were the most isolated organisms from a sample collected in Round 1 at 14.29% (2/14), followed by *E. coli*, *K. pneumoniae*, *Chryseobacterium* spp. and *E. falsenii* at 7.14% (1/14).

While chicken samples collected in Round 2, *C. indologenes* was the most isolated organism at 26.67% (4/15), followed by *Myroides* spp. and *S. multivorum* at 20% (3/15), and also detected *B. cereus* group, *B. thermoamylovorans*, *K. pneumoniae,* and *M. ordoratus* at 6.67% (1/15).

Whereas for chicken samples collected in Round 3, *E. brevis* was the most isolated organism at 21.43% (3/14), followed by *L. lactis* at 14.29% (2/14), and also detected *C. indologenes*, *Myroides* spp., and *S. multivorum* at 7.14% (1/14).

**Figure 7:**
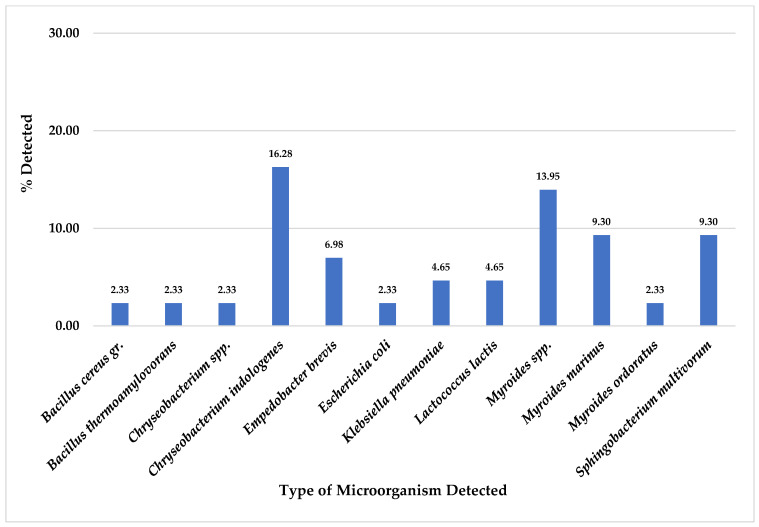
Total Percentage of Microorganisms Detected in Pork Meat Samples from Fresh Markets in Chiang Mai Province (September 2023–February 2024, *n* = 43). Microorganisms were isolated using CHROMID™ VRE agar plates.

For a total of 43 samples of pork meat collected, this study found *C. indologenes* was the most detected on CHROMID™ VRE agar plates at 16.28% (7/43), followed by *Myroides* spp. at 13.95% (6/43), *M. marinus* and *S. multivorum* at 9.30% (4/43), and *E. brevis* at 6.98% (3/43). Moreover, other organisms were also detected in pork meat samples at equal or less than 4.65% (2/43), as shown in Figure 7.

**Figure 8:**
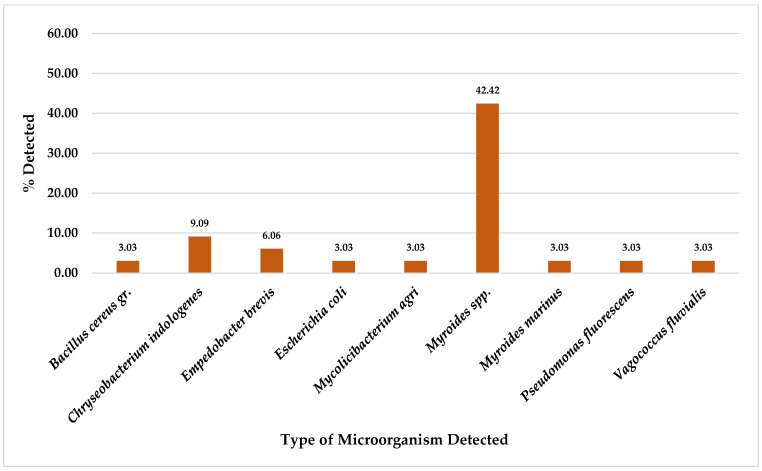
Total Percentage of Microorganisms Detected in Chicken Meat Samples from Fresh Markets in Chiang Mai Province (September 2023–February 2024, *n* = 33). Microorganisms were isolated using CHROMID™ VRE agar plates.

Of a total of 33 chicken meat collected samples, this study found *Myroides* spp. was the most detected on CHROMID^TM^ VRE agar plates at 42.42% (14/33), followed by *C. indologenes* at 9.09% (3/33) and *E. brevis at* 6.06% (2/33). Furthermore, other organisms were also detected in chicken meat samples at 3.03% (1/33), as shown in Figure 8.

### 3.2. Antimicrobial Susceptibility Test

From overall 43 raw pork meat and 33 chicken meat samples randomly collected from three markets in Chiang Mai province during September 2023–February 2024, this study found no suspected antibiotic-resistant microorganisms with specific-colored colonies related to Carbapenem-resistant *Enterobacteriaceae* (CRE), Methicillin-Resistant *Staphylococcus aureus* (MRSA) and Vancomycin-resistant *Enterococci* (VRE) bacteria detected.

Nevertheless, we detected 20 out of 43 (46.51%) *E. coli* and 4 of 43 (9.30%) of *K. pneumoniae* from pork meat samples and 23 of 33 (69.70%) of *E. coli* and 2 of 33 (6.06%) of *K. pneumoniae* from chicken meat samples isolated on CHROMID^®^ ESBL agar plates (bioMerieux SA, France). All these suspected specific-colored isolated colonies were used to confirm ESBL resistance with the antimicrobial drug susceptibility tests on Mueller-Hinton agar (MHA) plates using the disc diffusion Kirby-Bauer technique, and the results were interpreted according to the Standards for Antimicrobial Susceptibility of the Clinical Laboratory Standards Institute (CLSI) protocol.

Antibiotic discs with CAZ30, CTX30, CAC30/10, and CEC30/10 were used for these tests. The zones of inhibition were measured to the nearest millimeter at the back of the inverted culture plate to examine an antimicrobial drug susceptibility test; it was considered ESBL positive when an increase of ≥5 mm for the zone diameter of any of the antibiotic disks used towards the center [36].

The antimicrobial drug susceptibility test results found all 20 suspected colonies of *E. coli* and four suspected colonies of *K. pneumoniae* from pork meat samples, and all 23 suspected colonies of *E. coli* and two suspected colonies of *K. pneumoniae* from chicken meat samples were 100% resistant to ESBL.

### 3.3. Seasonal ESBL-Producing Organism Detection

**Figure 9:**
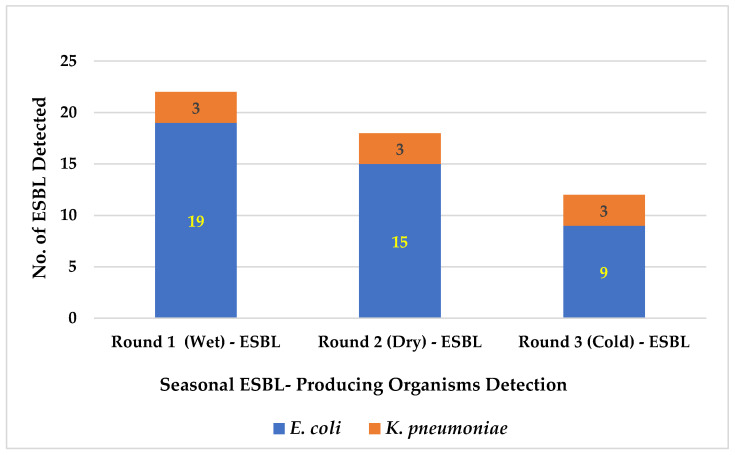
Total Number of ESBL-Producing Microorganisms Detected in Pork and Chicken Meat Samples Collected from Fresh Markets in Chiang Mai Province. The total number of ESBL-producing organisms and ESBL-producing *E. coli* significantly decreased across Round 1 (Wet/Rainy), Round 2 (Dry), and Round 3 (Cold) of meat sample collection (*p* = 0.040).

The results of overall ESBL-producing organisms detected in pork and chicken meat samples in this study are shown in Figure 9. This study found ESBL-producing organisms detected from 22, 18, and 12 of meat samples from Rounds 1, 2, and 3 of sample collection, respectively. For Round 1 of meat samples collected during the wet or rainy season, ESBL-producing *E. coli* and *K. pneumoniae* were detected from 19 and 3 of the meat samples, respectively. Whereas for Round 2, collected during the dry season, and Round 3, collected during the cold season, ESBL-producing *E. coli* and *K. pneumoniae* were detected as 15 and 3 for Rounds 2 and 9, and 3 for Round 3, respectively. Moreover, the results of statistical analysis by One-way ANOVA test found the total number of ESBL-producing organisms and ESBL-producing *E. coli* were significantly decreased from Rounds 1, 2, and 3 of meat samples collected (*p* = 0.040).

### 3.4. Ciprofloxacin Detections and Bacterial Contamination in Positive Samples

In this study, raw pork and chicken meat samples were used to determine ciprofloxacin antibiotics and to detect antibiotic-resistant organisms in these samples. Of a total of 76 samples (43 pork samples and 33 chicken samples), ciprofloxacin residue was detected in nine samples (11.84%) [6 (13.95%) pork samples and three (9.09%) chicken samples]. Bacteria isolation in ciprofloxacin-positive samples are shown in Table 5. From these positive samples, antibiotic-resistant organisms such as ESBL-producing *E. coli* bacteria were detected in one of six pork samples (16.67%) and two of three chicken samples (66.67%). Whereas other microorganisms were detected with significant diversity in pork samples, encompassing *Aeromonas* spp., *Chryseobacterium* spp., *C. indologenes*, *P. aeruginosa*, *Pseudomonas* spp., etc. While *Aeromonas* spp., *Myroides* spp., *Pseudomonas* spp., and other pathogens were also detected from ciprofloxacin-positive chicken samples.

## 4. Discussion

This study presents the results of antibiotic-resistant microorganisms contaminated and ciprofloxacin antibiotic residue detected in raw pork and chicken meat samples randomly purchased from three fresh markets in Chiang Mai Province, Northern Thailand during September 2023–January 2024. These three markets were chosen and given consideration because they are influenced by a large number of city dwellers. One of the three is the largest wholesale market, where food products and materials are distributed throughout the province and surrounding area, while the other two marketplaces are well-known and popular. Therefore, the findings of this study may have an impact on the local population.

Ciprofloxacin antibiotics are extensively utilized in poultry production for prophylactic and therapeutic purposes. The efficacy of immunoassay test kits for monitoring specific antibiotic residues in meat samples was evidenced. The affirmative identification in several meat samples raises alarms. Prolonged exposure to antibiotic leftovers in the body may result in acute or chronic organ damage and systemic harm. Their presence may potentially induce allergic reactions or foster the development of drug-resistant microorganisms in people following prolonged exposure. The positive samples also showed microbial, which has antibiotic resistance [29]. There was no study of ciprofloxacin in raw meat products in Thailand. However, ciprofloxacin was intensively used in poultry production and has allowed better treatment of several diseases; however, their prudent, ethical, and professional use is essential. The inherent risks of the inadequate use of antimicrobials in poultry production include the induction of bacterial resistance, environmental contamination, and the accumulation of residues in raw meat products [29].

Hence, there is a need to respect the withdrawal periods of antimicrobials in order to reduce the level of antimicrobial residues in meat samples to a minimum and also to reinforce controls through regular sampling and analysis. Further studies on the different types of antibiotics used in poultry, piggery, and cattle farms in Mafikeng are recommended.

For the results of antibiotic-resistant organisms detected in raw pork and chicken meat samples in this study, CHROMID^®^ culture media (bioMerieux SA, Marcy l’Etoile, France) was used in the microbial culture step. CHROMID^®^, or chromogenic media, is the innovative chromogenic media for rapid, accurate culture and identification of high-prevalence clinically relevant pathogen recovery, including multidrug-resistant organisms. These pathogens can be quickly and consistently detected and identified using the broad and inventive CHROMID^®^ range of chromogenic medium. Even with mixed cultures, CHROMID^®^’s colors’ intensity and specificity allow for easy identification and outcome. These colonies are also well isolated and easy to identify by differentiating colors with colorless backgrounds [37].

Generally, CHROMID^®^ ESBL media or chromogenic media for ESBL screening is based on agar with a patented mixture of antibiotics designed specifically to enable the selective growth of ESBL-producing *Enterobacteriaceae.* Chromogenic substrates allow for the immediate identification of the most encountered ESBL-producing *Enterobacteriaceae.* Whereas CHROMID^®^ CARBA is a selective chromogenic media for the isolation of Carbapenemase-producing *Enterobacteriaceae* (CPE) or Carbapenem-resistant *Enterobacteriaceae* (CRE), CHROMID^®^ MRSA is a selective chromogenic media that has been designed to produce green colonies for methicillin-resistant *S. aureus* (MRSA), these green colored colonies resulting from alpha-glucosidase producing colonies in the presence of an antibiotic, cefoxitin; and CHROMID^®^ VRE is chromogenic media for the rapid & reliable qualitative detection of *E. faecium* and *E. faecalis* showing acquired vancomycin resistance [38].

In the total 43 raw pork meat and 33 chicken meat samples, we found no antibiotic-resistant organisms related to CRE, MRSA, and VRE bacteria were detected. Nevertheless, the ESBL-producing bacteria were only detected in these samples: 20 of 43 (46.51%) of *E. coli* and 4 of 43 (9.30%) of *K. pneumoniae* from pork meat samples (Figure 1) and 23 of 33 (69.70%) of *E. coli* and 2 of 33 (6.06%) of *K. pneumoniae* from chicken meat samples (Figure 2) were isolated from CHROMID^®^ ESBL agar plates (bioMerieux SA, Marcy l’Etoile, France). All these suspected isolated colonies were 100% ESBL-producing bacteria confirmed by antimicrobial drug susceptibility tests. At the same time, another study from Thailand examined the occurrence and antimicrobial susceptibility of ESBL-producing *E. coli* isolates from 3 consumed minced meat varieties such as pork, chicken, and beef. They found that ESBL-producing *E. coli* was detected at 52%, and minced chicken meat was the most contaminated (79.17%) [39].

According to several studies, antibiotic resistance in bacterial pathogens is higher in poultry, pig, and other meat animals [18,19,20]. As a result, antibiotic-resistant strains from the gut may contaminate meat during slaughtering, processing, and transporting, and then resistant bacteria can infect consumers via meat [21,22,23].

*E. coli* and *K. pneumoniae* are two predominate organisms of the Gram-negative rod family (*Enterobacteriaceae*), which is a component of the normal found in the human gastrointestinal tract [40,41]. These are significant human pathogens that cause a broad range of hospital- and community-acquired infections, including meningitis, peritonitis, pneumonia, urinary tract infections (UTIs) [42], and septicemia [43,44]. Therefore, morbidities and mortalities will arise from these organisms’ infections if they are not treated appropriately [45].

Additionally, *E. coli* continues to be one of the most common causes of a number of bacterial diseases that affect both humans and animals, but it is more commonly linked to diarrhea in farm animals. Along with other significant bacterial foodborne agents like *Salmonella* spp. and *Campylobacter*, *E. coli* is regarded as the most significant human pathogen globally. It can cause severe infection [46].

ESBL is an enzyme found in Gram-negative rod-shaped bacteria that could degrade β-lactam drugs, including penicillin, oxyimino cephalosporin (3rd generation cephalosporin) and aztreonam, resulting in almost all β-lactam drug resistance. This enzyme is a new type of β-lactamase that has been found in humans, which is the most present and found worldwide since it compromises the effective treatment and is responsible for a high number of morbidity and mortality [47,48]. Due to their capacity to hydrolyze ß-lactam antibiotics, bacteria may be able to remain a source of infection and increase the overuse of antibiotics as a last option in human treatment [46].

There has been a global increase in the number of reports on ESBL-producing *E. coli* isolation from animals used in food production, primarily from chicken meat [47,49]. In this issue, the overuse of antibiotics in cattle has aided in the development of diseases resistant to antibiotics in humans. However, it has been noted that consuming meat, coming into close contact with infected animals, or environmental manure spreading are the ways in which antimicrobial-resistant infections are transferred from livestock to people [50,51]. Furthermore, in the digestive tracts of people and animals, gene transfer leading to antibiotic resistance may take place between distinct bacterial species [52].

Moreover, for the results of the seasonal ESBL-producing organism detected in this study, we found the total ESBL-producing organisms were highest detected in Round 1 of samples collected during the wet/rainy season and were significantly decreased in Round 2 collected in the dry season and Round 3 collected in the cold season, respectively (Figure 9). The result of some studies in Malawi found that the incidence of ESBL colonization varied over time, peaking in the wet season. The buildup of mud and floodwater, which could result in increased interaction with contaminated soil or water, which might lead to increased ESBL-producing organisms’ transmission, is one possible explanation [53]. Some research findings indicate that the spread of antibiotic resistance may be facilitated by rising temperatures linked to climate change, which could have an impact on a variety of ecosystems. Rivers, glaciers, soil, and medical environments all exhibit this behavior. Global health is at risk as rising temperatures are linked to an increase in the prevalence of antibiotic resistance in a variety of settings [54,55]. Furthermore, the relationship between AMR and climate varies according to the circumstances in various climate zones [56].

Even though this study could not detect any antibiotic-resistant microorganisms related to CRE, MRSA, and VRE organisms from 43 pork meat and 33 chicken meat samples, but we could detect several types of other organisms in these meat samples which were isolated from CHROMID^®^ media plates (Figure 1, Figure 2, Figure 3 and Figure 4).

From total 43 pork meat collected samples, for CHROMID^®^ ESBL media, *A. punctata (caviae)* and *P. putida* were the most detected (Figure 1); for CHROMID™ CARBA media, *A. sobria* was the most detected followed by *A. veronii* and *P. putida* (Figure 3); for CHROMID^®^ MRSA SMART media, *R. kristinae* was the most detected followed by *M. caseolyticus*, *V. fluvialis* and *E. faecalis* (Figure 5); and for CHROMID™ VRE media, *C. indologenes* was the most detected followed by *Myroides* spp., *M. marinus* and *S. multivorum* (Figure 7).

Whereas from a total of 33 chicken meat collected samples, for CHROMID^®^ ESBL media, *A. salmonicida*/*bestiarum* was the most detected, followed by *P. putida* and *A. punctata (caviae)* (Figure 2); for CHROMID™ CARBA media, *P. putida* was the most detected followed by *A. veronii*, *A. sobria* and *P. fluorescens* (Figure 4); for CHROMID^®^ MRSA SMART media, *M. caseolyticus* was the most detected followed by *E. faecalis* and *M. liquefaciens* (Figure 6); and for CHROMID™ VRE media, *Myroides* spp. was the most detected followed by *C. indologenes* and *E. brevis* (Figure 8).

Almost all of these microorganisms detected were opportunistic pathogens associated with various infections in immunocompromised and immunocompetent individuals. Normally, animal microbiome organisms, the atmosphere of the slaughterhouse, and the tools used before and after the slaughter process contaminate carcasses, the cuts that follow, and processed meat products. Certain bacterial contamination could proliferate or endure when food is being processed and stored, and also possible transmission of these pathogens to humans through the food chain [21,22,23].

The previous study reported poultry consumption, which was also shown to be the first cause of foodborne outbreaks in the USA between 1998 and 2012 [57]. Moreover, other emerging pathogens, such as *Aeromonas* spp., may also be considered. *Aeromonas* spp. are regarded as omnipresent, opportunistic, and main pathogens that are present in soil, aquatic settings, and a variety of raw meat products and vegetables. Almost all clinical *Aeromonas* isolates are from the three species *A. sobria*, *A. hydrophila*, and *A. veronii.* Most cases of *A. septicemia* include people with impaired immune systems. The two main risk factors for systemic infection are cancer and liver necrosis [58].

Whereas *C. indologenes* are aerobic, Gram-negative, and non-fermentative rods.1 It naturally exists in plants, water, soil, and food items. The human microbiota typically does not contain this organism [59,60]. Meningitis, bacteremia, pneumonia, myositis, keratitis, and indwelling devices are among the illnesses that have been reported. Within medical facilities, water systems and damp surfaces can harbor *C. indologenes*, which could act as a source of infection. It can endure municipal water systems and is resistant to chlorination [61].

*P. putida* is a rod-shaped, Gram-negative bacteria that is a member of the fluorescent pseudomonad family. Because of its remarkable resistance to difficult living circumstances, it is typically found on inert surfaces in hospitals and humid environments, where it can lead to nosocomial infections. It also frequently manifests in patients with invasive medical equipment, immunological dysfunction, or immunocompromised patients [62].

*E. faecalis* and *E. faecium* are the two most often isolated species from clinical samples. These Enterococci are opportunist pathogens that could cause nosocomial and community-acquired infections. One of the main reasons Enterococci can thrive in hospitals is due to their innate resistance to some of the frequent antibiotics used there and their capacity to develop resistance to them [63].

*R. kristinae* is a Gram-positive bacterium on the skin, oral and respiratory tract in humans. In immunocompromised people, it can lead to opportunistic infections associated with a number of severe diseases, such as septicemia, endocarditis, pneumonia, and peritonitis [64].

The genera *Myroides*, *Empedobacter*, and *Sphingobacterium* are ubiquitous in the environment. primarily of environmental origin (soil, plants, water, food, etc.) and have been isolated in moist areas within hospitals. Some strains can survive in chlorinated municipal water supplies, giving them a selective advantage in hospital water systems. Infections with *Elizabethkingia*, *Myroides*, *Empedobacter*, and *Sphingobacterium* are relatively rare, affecting primarily neonates or immunocompromised patients. Infections vary and include intraabdominal, wound, endocarditis, pneumonia, and septicemia, and can be associated with indwelling devices such as catheters [65,66,67,68].

Based on the results of this study, in the future study, we have a plan to expand the study to include additional food types, geographic areas, or contaminant types based on our initial findings. In addition, collaboration and networking with experts in food safety, microbiology, chemistry, and public health to engage with organizations that focus on food safety, food hygiene, and public health for support and resources.

## 5. Conclusions

In this study, ciprofloxacin antibiotic residue and antibiotic-resistant microorganisms were detected in raw pork and chicken meat samples collected from fresh markets in Chiang Mai, Northern Thailand. The presence of antibiotic residues and antibiotic-resistant organisms in animal food products, particularly meats, poses significant public health concerns due to the potential for antibiotic resistance and foodborne illnesses. This study’s findings can be used to disseminate knowledge to the health care unit and people who are involved in the food chain as slaughtering, processing, transporting, and storage of raw meats concern and must be aware of food safety and hygiene including potential risks and dangers of antibiotic-resistant and opportunistic pathogens infections while being touched or consume these food materials. These data will also be used to develop plans for the control and surveillance of antibiotic resistance of pathogens and control of the use of antibiotic drugs for the future treatment of bacterial infections, which support prevention, healthcare, and the public health system.

## Figures and Tables

**Table 1:** The percentage of microorganisms in pork and chicken meat samples isolated from CHROMID^®^ ESBL agar plates.

Results of Microorganism Identification by MALDI-TOF MS (VITEK^®^ MS, bioMerieux, Marcy-l’Étoile, France)		
Round 1 Collection	Round 2 Collection	Round 3 Collection
Pork; *n* = 14	Pork; *n* = 15	Pork; *n* = 14
***Escherichia coli* (78.57%)** ***Klebsiella pneumoniae* (14.29%)** *Acinetobacter nosocomialis* (14.29%) *Acinetobacter ursingii* (21.43%) *Acinetobacter guillouiae* (21.43%) *Aeromonas* spp. (7.14%) *Aeromonas punctata* (*caviae*) (28.57%) *Aeromonas salmonicida/bestiarum* (7.14%) *Chryseobacterium* spp. (7.14%) *Chryseobacterium indologenes* (7.14%) *Kluyvera intermedia* (7.14%) *Pseudomonas* spp. (7.14%) *Pseudomonas fragi* (14.29%) *Pseudomonas putida* (14.29%) *Pseudomonas mosselii* (7.14%) *Stenotrophomonas maltophilia* (7.14%)	***Escherichia coli* (40%)** ***Klebsiella pneumoniae* (13.33%)** *Acinetobacter baumannii* (6.67%) *Acinetobacter bereziniae* (6.67%) *Acinetobacter seifertii* (6.67%) *Acinetobacter johnsonii* (6.67%) *Acinetobacter pittii* (6.67%) *Acinetobacter ursingii* (33.33%) *Aeromonas hydrophilia* (6.67%) *Aeromonas punctata (caviae)* (20%) *Brucella* spp. (6.67%) *Citrobacter braakii* (6.67%) *Citrobacter freundii* (6.67%) *Chryseobacterium gleum* (6.67%) *Chryseobacterium indologenes* (40%) *Pseudomonas fragi* (6.67%) *Pseudomonas putida* (20%) *Pseudomonas fluorescens* (13.33%) *Serratia fonticola* (13.33%) *Sphingobacterium multivorum* (6.67%) *Stenotrophomonas rhizophila* (6.67%)	***Escherichia coli* (21.43%)** *Acinetobacter johnsonii* (7.14%) *Aeromonas punctata* (*caviae*) (14.29%) *Aeromonas salmonicida/bestiarum* (7.14%) *Citrobacter freundii* (21.43%) *Empedobacter brevis* (7.14%) *Kluyvera intermedia* (7.14%) *Myroides* spp. (7.14%) *Pseudomonas fragi* (7.14%) *Pseudomonas putida* (28.57%) *Pseudomonas aeruginosa* (7.14%) *Pseudomonas fluorescens* (14.29%) *Serratia fonticola* (7.14%)
**Chicken; *n* = 11**	**Chicken; *n* = 11**	**Chicken; *n* = 11**
***Escherichia coli* (72.73%)** ***Klebsiella pneumoniae* (9.09%)** *Acinetobacter ursingii* (9.09%) *Aeromonas* spp. (9.09%) *Aeromonas hydrophilia* (9.09%) *Aeromonas punctata* (*caviae*) (36.36%) *Aeromonas salmonicida/bestiarum* (36.36%) *Aeromonas media* (18.18%) *Chryseobacterium indologenes* (9.09%) *Myroides* spp. (9.09%) *Pluralibacter gergoviae* (9.09%) *Pseudomonas* spp. (18.18%) *Pseudomonas putida* (36.36%) *Pseudomonas mosselii* (9.09%) *Serratia fonticola* (18.18%)	***Escherichia coli* (72.73%)** ***Klebsiella pneumoniae* (9.09%)** *Acinetobacter pittii* (18.18%) *Acinetobacter ursingi* (9.09%) *Aeromonas punctata* (*caviae*) (27.27%) *Aeromonas salmonicida/bestiarum* (18.18%) *Chryseobacterium indologenes* (18.18%) *Myroides* spp. (27.27%) *Pseudomonas fragi* (18.18%) *Pseudomonas putida* (27.27%) *Pseudomonas mosselii* (9.09%) *Pseudomonas fluorescens* (18.18%) *Serratia fonticola* (9.09%)	***Escherichia coli* (54.55%)** *Aeromonas salmonicida/bestiarum* (36.36%) *Citrobacter freundii* (9.09%) *Chryseobacterium indologenes* (9.09%) *Ewingella americana* (18.18%) *Lelliottia amnigena* (9.09%) *Pseudomonas fragi* (18.18%) *Pseudomonas putida* (18.18%) *Rahnella aquatilis* (9.09%) *Serratia fonticola* (9.09%) *Stenotrophomonas maltophilia* (18.18%)

**Table 2:** The percentage of microorganisms in pork and chicken meat samples isolated from CHROMID™ CARBA agar plates.

Results of Microorganism Identification by MALDI-TOF MS (VITEK^®^ MS, bioMerieux, Marcy-l’Étoile, France)		
Round 1 Collection	Round 2 Collection	Round 3 Collection
Pork; *n* = 14	Pork; *n* = 15	Pork; *n* = 14
*Aeromonas* spp. (28.57%) *Aeromonas veronii* (14.29%) *Aeromonas sobria* (78.57%) *Pseudomonas* spp. (14.29%) *Pseudomonas fragi* (14.29%) *Pseudomonas putida* (35.71%) *Pseudomonas aeruginosa* (7.14%) *Pseudomonas fluorescens* (28.57%) *Sphingobacterium multivorum* (7.14%) *Stenotrophomonas maltophilia* (7.14%)	*Aeromonas salmonicida*/*bestiarum* (20%) *Aeromonas veronii* (73.33%) *Aeromonas sobria* (20%) *Pseudomonas* spp. (6.67%) *Pseudomonas fragi* (20%) *Pseudomonas putida* (46.67%) *Pseudomonas mosselii* (6.67%) *Pseudomonas fluorescens* (6.67%) *Pseudomonas rhodesiae* (6.67%) *Sphingobacterium multivorum* (40%) *Stenotrophomonas maltophilia* (26.67%)	*Acinetobacter haemolyticus* (7.14%) *Aeromonas salmonicida/bestiarum* (7.14%) *Aeromonas media* (7.14%) *Aeromonas veronii* (35.71%) *Aeromonas sobria* (35.71%) *Chryseobacterium indologenes* (7.14%) *Hafnia alvei* (7.14%) *Pseudomonas fragi* (21.43%) *Pseudomonas putida* (35.71%) *Pseudomonas mosselii* (14.29%) *Pseudomonas fluorescens* (7.14%) *Pseudomonas veronii* (7.14%) *Serratia grimesii* (7.14%) *Serratia liquefaciens* (14.29%) *Sphingobacterium multivorum* (21.43%) *Stenotrophomonas rhizophila* (7.14%)
**Chicken; *n* = 11**	**Chicken; *n* = 11**	**Chicken; *n* = 11**
*Aeromonas* spp. (18.18%) *Aeromonas veronii* (18.18%) *Aeromonas sobria* (81.82%) *Pseudomonas* spp. (27.27%) *Pseudomonas fragi* (18.18%) *Pseudomonas putida* (63.64%) *Pseudomonas mosselii* (18.18%) *Pseudomonas fluorescens* (27.27%)	*Aeromonas salmonicida*/*bestiarum* (18.18%) *Aeromonas veronii* (63.64%) *Aeromonas sobria* (27.27%) *Chryseobacterium indologenes* (9.09%) *Pseudomonas* spp. (18.18%) *Pseudomonas fragi* (9.09%) *Pseudomonas putida* (54.55%) *Pseudomonas mosselii* (9.09%) *Pseudomonas fluorescens* (18.18%) *Stenotrophomonas maltophilia* (9.09%)	*Acinetobacter pittii* (9.09%) *Aeromonas salmonicida/bestiarum* (36.36%) *Aeromonas veronii* (63.64%) *Aeromonas sobria* (27.27%) *Pseudomonas chlororaphis* (9.09%) *Pseudomonas fragi* (18.18%) *Pseudomonas putida* (36.36%) *Pseudomonas aeruginosa* (9.09%) *Pseudomonas fluorescens* (36.36%) *Serratia liquefaciens* (9.09%) *Sphingobacterium multivorum* (9.09%) *Stenotrophomonas maltophilia* (18.18%) *Stenotrophomonas rhizophila* (9.09%)

**Table 3:** The percentage of microorganisms in pork and chicken meat samples isolated from CHROMID^®^ MRSA SMART agar plates.

Results of Microorganism Identification by MALDI-TOF MS (VITEK^®^ MS, bioMerieux, Marcy-l’Étoile, France)		
Round 1 Collection	Round 2 Collection	Round 3 Collection
Pork; *n* = 14	Pork; *n* = 15	Pork; *n* = 14
*Macrococcus caseolyticus* (21.43%) *Rothia kristinae* (14.29%) *Vagococcus fluvialis* (21.43%)	*Bacillus cereus* gr. (6.67%) *Cedecea neteri* (6.67%) *Macrococcus caseolyticus* (20%) *Microbacterium liquefaciens* (13.33%) *Rothia kristinae* (60%) *Vagococcus fluvialis* (13.33%)	*Carnobacterium divergens* (14.29%) *Cupriavidus gilardii* (7.14%) *Enterococcus faecalis* (28.57%) *Lactococcus garvieae* (14.29%) *Macrococcus caseolyticus* (21.43%) *Microbacterium liquefaciens* (7.14%) *Morganella morganii* (7.14%) *Rothia kristinae* (28.57%) *Staphylococcus saprophyticus* (7.14%) *Staphylococcus sciuri* (14.29%) *Vagococcus fluvialis* (21.43%) *Vibrio harveyi* (7.14%) *Weissella paramesenteroides* (14.29%)
**Chicken; *n* = 11**	**Chicken; *n* = 11**	**Chicken; *n* = 11**
*Lactococcus raffinolactis* (9.09%) *Macrococcus caseolyticus* (18.18%) *Microbacterium liquefaciens* (9.09%) *Vagococcus fluvialis* (9.09%)	*Cedecea davisae* (18.18%) *Corynebacterium pseudodiphtheriticum* (9.09%) *Enterococcus faecalis* (9.09%) *Lactococcus raffinolactis* (9.09%) *Macrococcus caseolyticus* (36.36%) *Microbacterium liquefaciens* (18.18%) *Rothia kristinae* (18.18%)	*Cedecea neteri* (9.09%) *Enterococcus faecalis* (36.36%) *Enterococcus faecium* (9.09%) *Macrococcus caseolyticus* (27.27%) *Microbacterium liquefaciens* (9.09%) *Microbacterium oxydans* (9.09%) *Rothia kristinae* (9.09%) *Staphylococcus kloosii* (9.09%) *Staphylococcus saprophyticus* (9.09%) *Staphylococcus sciuri* (18.18%) *Vagococcus fluvialis* (9.09%)

**Table 4:** The percentage of microorganisms in pork and chicken meat samples isolated from CHROMID™ VRE agar plates.

Results of Microorganism Identification by MALDI-TOF MS (VITEK^®^ MS, bioMerieux, Marcy-l’Étoile, France)		
Round 1 Collection	Round 2 Collection	Round 3 Collection
Pork; *n* = 14	Pork; *n* = 15	Pork; *n* = 14
*Chryseobacterium* spp. (7.14%) *Chryseobacterium indologenes* (14.29%) *Enterobacter falsenii* (7.14%) *Escherichia coli* (7.14%) *Klebsiella pneumoniae* (7.14%) *Myroides* spp. (14.29%) *Myroides marinus* (14.29%)	*Bacillus cereus* gr. (6.67%) *Bacillus thermoamylovorans* (6.67%) *Chryseobacterium indologenes* (26.67%) *Klebsiella pneumoniae* (6.67%) *Myroides* spp. (20%) *Myroides marinus* (13.33%) *Myroides ordoratus* (6.67%) *Sphingobacterium multivorum* (20%)	*Chryseobacterium indologenes* (7.14%) *Empedobacter brevis* (21.43%) *Lactococcus lactis* (14.29%) *Myroides* spp. (7.14%) *Sphingobacterium multivorum* (7.14%)
**Chicken; *n* = 11**	**Chicken; *n* = 11**	**Chicken; *n* = 11**
*Aeromonas* spp. (9.09%) *Aeromonas hydrophilia* (9.09%) *Aeromonas punctata (caviae)* (9.09%) *Aeromonas salmonicida*/*bestiarum* (18.18%) *Aeromonas media* (9.09%) *Chryseobacterium indologenes* (9.09%) *Escherichia coli* (36.36%) *Myroides* spp. (36.36%) *Pluralibacter gergoviae* (9.09%) *Pseudomonas* spp. (9.09%) *Pseudomonas putida* (27.27%) *Serratia fonticola* (9.09%)	*Bacillus cereus* gr. (9.09%) *Chryseobacterium indologenes* (9.09%) *Empedobacter brevis* (9.09%) *Escherichia coli* (9.09%) *Myroides* spp. (63.64%) *Myroides marinus* (9.09%) *Pseudomonas fluorescens* (9.09%) *Vagococcus fluvialis* (9.09%)	*Chryseobacterium indologenes* (9.09%) *Empedobacter brevis* (9.09%) *Mycolicibacterium agri* (9.09%) *Myroides* spp. (18.18%)

**Table 5:** This table shows the results of ciprofloxacin residue determination in pork and chicken meat samples and the microorganism contamination in these positive samples.

Samples	Positive Detection (%)	Code	Type of Microorganism Detected from CHROMID^®^ Selective Media			
			CHROMID^®^ ESBL	CHROMID™ CARBA	CHROMID^®^ MRSA	CHROMID™ VRE
Pork (*n* = 43)	6 (13.95)	P003	*Pseumonas* spp. *Chryseobacterium indologenes*	*Pseudomonas aeruginosa*	No growth	*Chryseobacterium* spp. *Enterobacter falsenii* *Empedobacter falsenii*
		P009	*Acinetibacter ursingii* *Pseudomanas fragi*	No growth	No growth	*Chryseobacterium indologenes*
		P010	*Pseudomonas mosselii*	*Aeromonas sobria*	No growth	No growth
		P023	***E. coli (ESBL)*** *Pseudomonas fluorescens* *Serratia fonticola*	*Pseudomonas fragi* *Pseumonas fluorescens* *Aeromonas veronii*	*Rothia kristinae*	*Sphingobacterium multivorum*
		P035	*Citrobacter freundii*	*Serratia liquefaciens*	*Macrococcus caseolyticus*	No growth
		P036	*Aeromonas salmonicida*/*bestiarum* *Kluyvera intermedia*	*Hafnia alvei*	No growth	*Empedobacter brevis* *Lactococcus lactis*
Chicken (*n* = 33)	3 (9.09)	C019	*Myroides* spp. *Aeromonas punctata (caviae)*	*Aeromonas veronii* *Pseudomonas putida*	*Lactococcus raffinolactis* *Rothia kristinae*	*Myroides* spp.
		C020	***E. coli (ESBL)*** *Myroides* spp. *Acinetobacter pittii*	*Aeromonas veronii*	*Rothia kristinae*	*Myroides* spp. *Pseumonas fluorescens*
		C029	***E. coli (ESBL)*** *Aeromonas salmonicida/bestiarum*	*Aeromonas salmonicida*/*bestiarum* *Aeromonas veronii* *Pseudomanas fragi* *Pseudomonas putida* *Pseudomonas fluorescens* *Stenotrophomonas maltophilia*	*Enterococcus faecalis* *Staphylococcus saprophyticus*	No growth

## Data Availability

The original contributions presented in this study are included in the article. Further inquiries can be directed to the corresponding author.
